# Effect of immune checkpoint inhibitor time-of-day infusion on survival in advanced biliary tract cancer: a propensity score-matched analysis

**DOI:** 10.3389/fimmu.2024.1512972

**Published:** 2024-12-18

**Authors:** Yichen Zheng, Fanfan Shi, Lingqi Sun, Jiamin Guo, Tonghui Ren, Ji Ma

**Affiliations:** ^1^ Department of Medical Oncology, Cancer Center and Laboratory of Molecular Targeted Therapy in Oncology, West China Hospital, Sichuan University, Chengdu, Sichuan, China; ^2^ Department of Clinical Research and Management, Center of Biostatistics, Design, Measurement and Evaluation (CBDME), West China Hospital, Sichuan University, Chengdu, Sichuan, China; ^3^ Sleep Medicine Center, Mental Health Center, West China Hospital, Sichuan University, Chengdu, China

**Keywords:** immune checkpoint inhibitor, biliary tract cancer, chronotherapy, circadian, propensity score-matched analysis

## Abstract

**Background:**

Circadian rhythms in the immune system and anti-tumor responses are underexplored in cancer immunotherapy. Despite the success of immune checkpoint inhibitors (ICIs) in treating advanced biliary tract cancers (BTCs), not all patients benefit. This study examined whether the timing of ICI administration affects outcomes in advanced BTC patients.

**Methods:**

We included advanced BTC patients from West China Hospital of Sichuan University who received ≥2 ICI treatments from October 2019 to September 2023, with follow-up until May 2024. Primary outcome was overall survival (OS), with secondary outcomes including progression-free survival (PFS), objective response rate (ORR), and adverse events (AEs). Propensity score matching (1:2 ratio, caliper width 0.1) mitigated confounding factors. Cox proportional hazards regression analyzed the impact of ICI timing (post-16:30) on OS and PFS. Chi-square test assessed ORR and AE differences.

**Results:**

Among 221 patients, 51 received ≥20% of ICIs after 16:30; 170 received <20%. Post-matching, 49 late-infusion patients had significantly shorter OS (median 10.1 *vs*. 14.5 months, HR=1.80, P=0.012) compared to 90 early-infusion patients. Pre-matching, late-infusion patients also had shorter OS (median 9.8 *vs*. 13.7 months, HR=1.68, P=0.010) and PFS (median 4.9 *vs*. 8.1 months, HR=1.62, P=0.006). Multivariate analysis confirmed these results. No significant differences were found in ORR (χ^2 = 1.53, P=0.215) or AEs (all P>0.050). Sensitivity analyses supported these findings.

**Conclusion:**

Timing of ICI administration affects efficacy in advanced BTC, with pre-16:30 infusions linked to better survival. Larger, prospective studies are needed to validate these results.

## 1 Introduction

Biliary tract cancers (BTCs), aggressive tumors originating from the epithelial cells of the bile ducts, are categorized anatomically into gallbladder cancer, perihilar cholangiocarcinoma, distal cholangiocarcinoma, and intrahepatic cholangiocarcinoma. Despite their global rarity—constituting less than 1% of all cancers—the incidence of BTCs is increasing (1). These cancers are highly malignant and prognostically unfavorable, with a 5-year OS rate below 20% (1). BTC typically presents insidiously, with 60-70% of patients diagnosed with unresectable disease. Even those undergoing potentially curative surgery face high recurrence rates, between 70% and 75% (2). Historically, the combination chemotherapy of gemcitabine and cisplatin, validated by the ABC-02 study, served as the standard first-line treatment for advanced BTC (3). However, its efficacy has been unsatisfactory. Recent breakthroughs in immunotherapy have revolutionized first-line treatment options. The TOPAZ-1 study demonstrated that adding durvalumab to the gemcitabine-cisplatin regimen significantly improves OS (4). Similarly, the KEYNOTE-966 study confirmed enhanced survival with the addition of pembrolizumab (5). Despite these advancements, the response to immunotherapy remains limited to a subset of patients. Predictive biomarkers such as programmed death-ligand 1 (PD-L1) expression, microsatellite instability-high, and tumor mutational burden provide some guidance for immunotherapy selection, but their predictive accuracy is limited (6, 7). Challenges such as the invasiveness of obtaining samples, intratumoral heterogeneity, the high cost of tests like immunohistochemistry and next-generation sequencing, and frequently inadequate biopsy specimens restrict their utility. Thus, the development of more convenient, accurate, and cost-effective predictive markers remains a critical need in advancing the treatment of BTC.

The circadian rhythmicity of the immune system is a critical factor that has traditionally been overlooked in cancer immunotherapy. Recent evidence increasingly supports the notion that the efficacy of immunotherapy might vary diurnally. For example, studies have demonstrated that the daytime administration of interferon-β yields a stronger antitumor response in mice compared to evening dosing (8). Clinical evidence also supports this circadian influence. A trial with stage I-II renal cell carcinoma patients revealed that IL-2 administered in the early morning (5:00–13:00) or late night (21:00–5:00) resulted in longer survival compared to afternoon administration (13:00–21:00) (9). In the context of immune checkpoint inhibitors (ICIs), the MEMOIR study pioneered the finding that administering ICIs earlier in the day (with less than 20% of infusions after 16:30) improved prognosis in melanoma patients (10). This suggests that the timing of ICI administration could significantly impact the effectiveness of immunotherapy. Although similar patterns have been observed in retrospective studies on non-small cell lung cancer and renal cell carcinoma (11–13), a pan-cancer study encompassing various solid tumors did not produce significant findings (14). This discrepancy could be attributed to the inclusion of tumors with lower immunogenicity in the pan-cancer study, whereas positive results have primarily been reported in cancers that are more responsive to immunotherapy (10–13, 15–17). Moreover, a recent meta-analysis involving 1,663 patients further supported these findings (18). It demonstrated a significant association between earlier ICI administration and improved OS and PFS, providing additional evidence for the value of chronotherapy in immunotherapy.

BTC is characterized by a poor prognosis, underscoring the urgent need to enhance the efficacy of available treatments, including immunotherapy. Historically regarded as an “immune cold” tumor (19–21), the application of immunotherapy in BTC is a recent development and remains limited in clinical experience. Given this context, it is imperative to explore novel factors that might influence the effectiveness of these treatments. One such factor is the timing of ICI administration. To investigate whether the timing of ICI infusions affects clinical outcomes in advanced BTC, we conducted a single-center retrospective study. We utilized propensity score matching and multivariate Cox proportional hazards regression analyses to minimize potential biases and ensure the robustness of our findings.

## 2 Materials and methods

### 2.1 Study population

Our study included patients with BTC treated at West China Hospital, Sichuan University, from October 2019 to September 2023, with follow-up data available up to May 31, 2024. We enrolled patients who received at least two infusions of ICIs. Data on clinical and pathological characteristics, timing of ICI infusions, and survival outcomes were extracted from the electronic medical records of West China Hospital and supplemented by telephone follow-ups. The inclusion criteria for the study were: (1) histologically confirmed BTC; (2) presence of inoperable or advanced disease at the initiation of immunotherapy; (3) receipt of at least two cycles of ICIs, either as monotherapy or in combination with chemotherapy or targeted therapy. Patients were excluded if they had incomplete medical records. This study received ethical approval from the Ethics Committee of West China Hospital, Sichuan University (Approval No. 2023699).

### 2.2 Procedures

The administration of ICIs, chemotherapy, and targeted therapy was tailored by the attending oncologists based on individual patient needs; thus, the doses and cycles were not standardized in advance. However, initial dosing for ICIs adhered to the following standard protocols: durvalumab at 1500 mg, sintilimab, pembrolizumab, and camrelizumab each at 200 mg, and toripalimab at 240 mg, all administered every three weeks. Other ICIs such as envafolimab and tislelizumab, utilized by a minority of patients, are not discussed in detail in this document. Doses of chemotherapy and targeted therapies were subject to adjustments depending on the patient’s tolerance and overall health status. Imaging follow-ups were generally conducted after every 2-3 treatment cycles to assess tumor response, which was evaluated using the Response Evaluation Criteria in Solid Tumors (RECIST) version 1.1 (22).

AEs were retrospectively assessed from the initiation of treatment until the last follow-up, using the medical records. AEs were documented according to the Common Terminology Criteria for Adverse Events (CTCAE) version 5.0 (23). Distinctions between immunotherapy-related adverse events (irAEs) and general AEs are not yet well-defined, commonly including symptoms such as rashes, thyroid dysfunctions, immune hepatitis, and immune myocarditis. For our analysis, irAEs were categorized following the methodology used in the TOPAZ-1 study (4).

Previous research has identified 16:30 as a significant time point that could potentially influence the efficacy of ICI treatment (10, 12). Accordingly, we adopted 16:30 as a cutoff time to analyze its impact on treatment outcomes. For sensitivity analysis, we further explored the outcomes using alternative cutoff times of 16:00 and 15:30.

### 2.3 Outcome measures

The primary outcome of this study is OS, defined as the time from the initiation of immunotherapy to the death of the patient. Secondary outcomes include PFS, ORR, and the incidence of AEs. PFS is determined as the duration from the start of immunotherapy to either tumor progression or death, whichever occurs first. Patients lost to follow-up were censored at their last known contact. ORR represents the proportion of patients achieving either a complete response or a partial response, as defined by the RECIST version 1.1 criteria. AEs were assessed from the initiation of ICI treatment to the last follow-up and were graded according to the CTCAE version 5.0. The incidence of specific irAEs, such as hypothyroidism, rash, and cardiac events, was separately recorded, utilizing the categorization criteria from the TOPAZ-1 study.

### 2.4 Statistical methods

In alignment with the MEMOIR study (10), we designated 16:30 as the cutoff time, dividing our study sample into two groups: patients who received ≥20% of their ICI infusions after 16:30, and those who received <20% after this time. We selected a 20% cutoff for the proportion of infusions as it is consistent with similar studies (10, 12, 13, 15), facilitating comparisons and clinical implementation. A lower cutoff, such as 10%, would categorize most patients into the later infusion group, whereas a higher cutoff would result in too few patients in that group.

Initially, we undertook data cleaning and variable processing. Given that the pre-treatment values for Carbohydrate Antigen 19-9 (CA19-9), Carcinoembryonic Antigen (CEA), Carbohydrate Antigen 125 (CA125), and Neutrophil-Lymphocyte Ratio (NLR) were missing in fewer than 5% of cases and were not normally distributed, we imputed missing data using median values and dichotomized these variables for analysis. We described patient characteristics between groups with continuous variables presented as either mean ± standard deviation or median (interquartile range), and categorical variables as frequencies and percentages. For assessing the balance of characteristics between groups, we utilized the χ² test or Fisher’s exact test for categorical variables and the t-test for the continuous variable, age. To enhance comparability between infusion groups, we performed 1:2 propensity score matching using logistic regression, setting a caliper of 0.1 (the maximum allowable difference in propensity scores). Although equal ratio matching is sometimes considered more persuasive, the disparity in group sizes justified using a 1:2 matching ratio to better utilize available data. We conducted univariate Cox proportional hazards regression to identify factors potentially affecting OS with a significance level set at p < 0.10 (results detailed in **Supplementary Table S2**). These factors included site of origin (intrahepatic *vs*. perihilar *vs*. distal *vs*. gallbladder), degree of differentiation (poorly *vs*. moderately-to-well), type of ICI (Anti-programmed cell death 1 (PD-1) *vs*. Anti-PD-L1), line of treatment for ICI (first-line *vs*. ≥2 lines), pre-treatment CA19-9 < 500 U/mL, pre-treatment CEA < 5 ng/mL, pre-treatment CA125 < 28.65 U/mL, and smoking status. Post-matching, we assessed the balance of characteristics between the matched groups.

We employed the Kaplan-Meier method to calculate OS and the associated 95% confidence intervals (CIs) for both unmatched and matched cohorts. Univariate Cox proportional hazards regression was utilized to estimate the unadjusted hazard ratios (HRs) for OS, along with their 95% CIs. For a more comprehensive analysis, multivariate Cox proportional hazards regression was applied to compute adjusted HRs and 95% CIs after accounting for factors that could influence OS. For secondary outcomes, which were assessed only in the unmatched cohort, both unadjusted and adjusted HRs for PFS with 95% CIs were calculated using univariate and multivariate Cox proportional hazards regression methods. Differences in ORR and the incidence of AEs between the groups were analyzed using the χ² test or Fisher’s exact test, depending on the data distribution.

Additionally, we conducted sensitivity analyses to explore the impact of time-of-day infusion on survival outcomes, focusing on populations with ≥2 ICI infusions at cut-off times of 15:30 and 16:00. To address potential biases due to insufficient observations, spurious infusion patterns, and other confounding factors, further analyses were extended to populations with ≥3 ICI infusions at 15:30, 16:00, and 16:30. These analyses were designed to verify the stability of the association between survival outcomes and time-of-day infusion, both in terms of magnitude and direction.

## 3 Results

**Figure 1:**
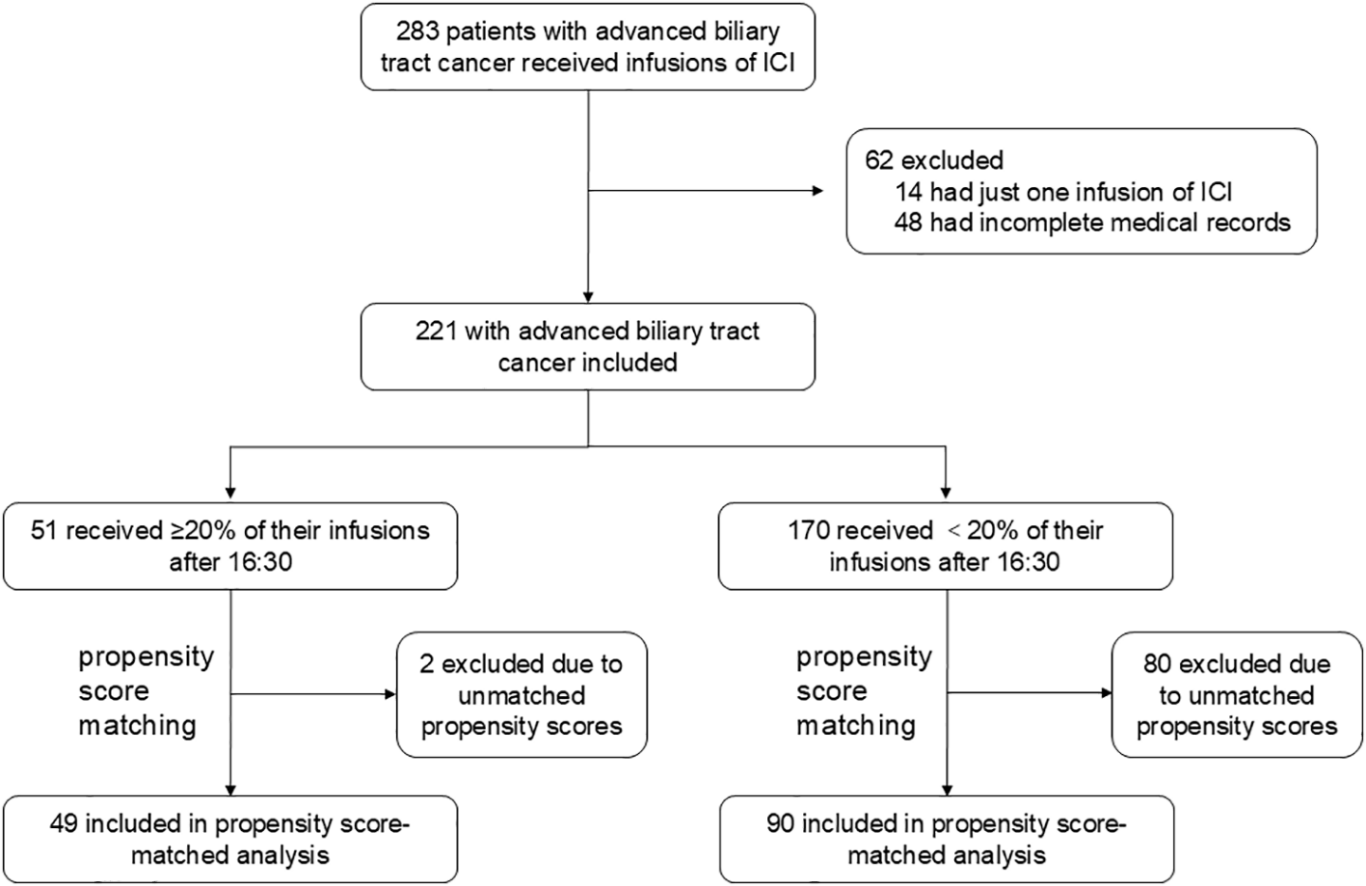
Study profile A total of 283 patients with advanced biliary tract cancer received infusions of immune checkpoint inhibitors (ICIs) between October 2019 and September 2023. After excluding 14 patients with only one infusion and 48 patients with incomplete medical records, 221 patients were included in the study cohort. Propensity score matching (1:2 ratio, caliper width = 0.1) was conducted based on key variables, including site of origin (intrahepatic *vs*. extrahepatic *vs*. gallbladder), degree of differentiation (poorly *vs*. moderately-to-well), type of ICI (Anti-PD-1 *vs*. Anti-PD-L1), line of treatment (first-line *vs*. ≥2 lines), pre-treatment levels of CA19-9 (< 500 U/mL), CEA (< 5 ng/mL), CA125 (< 28.65 U/mL), and smoking status. This process resulted in a matched cohort of 139 patients, comprising 49 who received ≥ 20% of their infusions after 16:30 and 90 who received < 20%. The remaining 82 patients were excluded due to unmatched propensity scores, with 1 patient from the ≥ 20% group and 80 patients from the < 20% group. CA125, Carbohydrate Antigen 125; CA19-9, Carbohydrate Antigen 19-9; CEA, Carcinoembryonic Antigen; ICI, immune checkpoint inhibitor; PD-1, programmed cell death 1; PD-L1, programmed death-ligand 1.

From the cohort criteria, 221 patients with ≥2 ICI infusions were included as the pre-matched cohort (**Figure 1**), of which 178 patients (81%) received ≥3 ICI infusions. The average age was 57.97 ± 10.26 years, with 117 males (53%). ICIs were used as first-line therapy in 137 patients (62%). Among the 221 patients, 172 (78%) were treated with a combination of ICI and chemotherapy, while 19 (9%) received a regimen comprising ICI, chemotherapy, and anti-angiogenic drugs. Five patients (2%) underwent ICI monotherapy, and 21 (10%) were treated with a combination of ICI and anti-angiogenic drugs. A small number of patients followed alternative regimens: one patient (<1%) received ICI, chemotherapy, and targeted therapy (Trastuzumab); another (<1%) was treated with ICI, anti-angiogenic drugs, and targeted therapy (Cetuximab); and two patients (<1%) received ICI and targeted therapy (Trastuzumab). Primary tumor types included intrahepatic bile duct cancer (64%), perihilar bile duct cancer (12%), distal bile duct cancer (7%), and gallbladder cancer (17%). A total of 51 patients (23%) had ≥20% of ICI infusions after 16:30, while 170 (77%) had <20% after this time. Baseline characteristics are detailed in **Supplementary Table S1**. Following the identification of influential factors via univariate Cox proportional hazards regression (detailed in **Supplementary Table S2**), a 1:2 propensity score matching yielded a cohort of 139 patients, with 49 (35%) receiving ≥20% of infusions after 16:30 and 90 (65%) receiving <20%. The baseline characteristics of the matched samples are presented in **Supplementary Table S1**.

**Figure 2:**
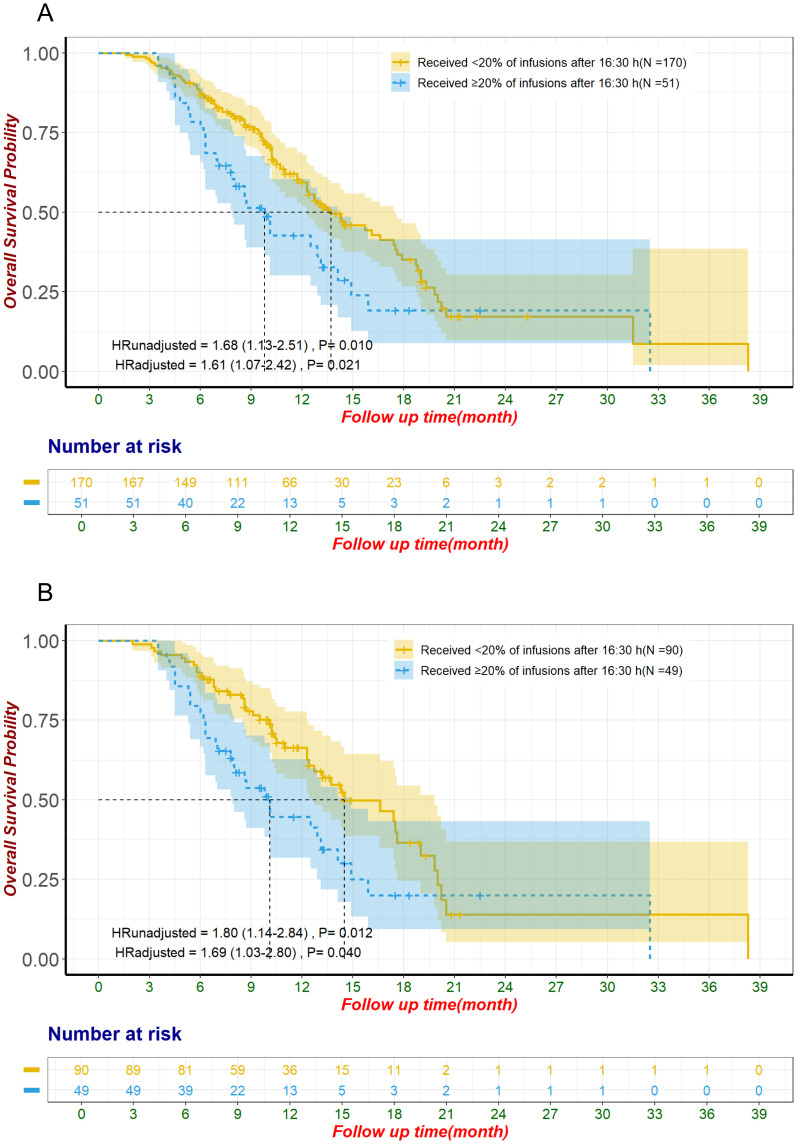
Overall survival for unmatched groups **(A)** and propensity score-matched groups **(B)** The adjustment factors include site of origin, degree of differentiation, type of ICI, line of treatment for ICI, pre-treatment CA19-9 level less than 500 U/mL, pre-treatment CEA level less than 5 ng/mL, pre-treatment CA125 level less than 28.65 U/mL, smoking status. CA125, Carbohydrate Antigen 125; CA19-9, Carbohydrate Antigen 19-9; CEA, Carcinoembryonic Antigen; ICI, immune checkpoint inhibitor.

In the pre-matched cohort, patients with ≥20% of ICI infusions after 16:30 had a median follow-up of 8.3 months, compared to 10.45 months for those with <20% late infusions. Kaplan-Meier analysis showed significantly reduced OS for patients with ≥20% late infusions, with a median OS of 9.8 months (95% CI: 7.9–14.1) versus 13.7 months (95% CI: 12.3–17.5) for those with fewer late infusions (HR: 1.68, 95% CI: 1.13–2.51, p = 0.010; **Figure 2A**).

After propensity score matching, patients with ≥20% of ICI infusions after 16:30 had significantly shorter OS, with a median of 10.1 months (95% CI: 7.9–14.9) compared to 14.5 months (95% CI: 12.7–19.8) for those with fewer late infusions (HR: 1.80, 95% CI: 1.14–2.84, p = 0.012; **Figure 2B**). This association remained significant in the multivariate Cox proportional hazards regression analysis, after adjusting for factors including site of origin (intrahepatic *vs*. perihilar *vs*. distal *vs*. gallbladder), degree of differentiation (poorly *vs*. moderately-to-well), type of ICI (Anti-PD-1 *vs*. Anti-PD-L1), line of treatment for ICI (first-line *vs*. ≥2 lines), pre-treatment CA19-9 ≤ 500 U/mL, pre-treatment CEA ≤ 5 ng/mL, pre-treatment CA125 < 28.65 U/mL, and smoking status. The adjusted HR was 1.69 (95% CI: 1.03-2.80, P=0.040; **Table 1**, **Figure 2B**). Analysis of the entire unmatched sample through multivariate Cox proportional hazards regression also corroborated these findings, with an HR of 1.61 (95% CI: 1.07-2.42, P=0.021; **Figure 2A**).

**Table 1:** Multivariable cox proportional hazards regression of overall survival, in the propensity score-matched patients who did (n=49) and did not (n=90) receive ≥20% of infusions after 1630 h.

Variable	Overall Survival hazard ratio (95% CI)	*P* value
Received ≥20% infusions after 1630 h	1.69 (1.03-2.80)	0.040*
Site of origin (Reference=Intrahepatic)		
Perihilar	1.52 (0.74-3.13)	0.253
Distal	0.52 (0.10-2.76)	0.444
Gallbladder	1.03 (0.52-2.06)	0.923
Degree of differentiation (Reference=Poorly)		
Moderately-to-well	0.45 (0.26-0.77)	0.004*
Type of ICI (Reference=Anti-PD-1)		
Anti-PD-L1	0.69 (0.37-1.31)	0.260
Line of treatment for ICI (Reference=First line)		
≥2 lines	1.99 (1.13-3.51)	0.018*
Pre-treatment CA19-9 <500 U/mL	0.29 (0.17-0.52)	<0.001**
Pre-treatment CEA < 5 ng/mL	1.10 (0.63-1.90)	0.740
Pre-treatment CA125<28.65 U/mL	0.54 (0.30-0.98)	0.044*
Former/Current smoke	0.90 (0.46-1.76)	0.748

ICI, immune checkpoint inhibitor; CA19-9, Carbohydrate Antigen 19-9;CEA, Carcinoembryonic Antigen;CA125, Carbohydrate Antigen 125; NLR, Neutrophil-to-Lymphocyte Ratio.

*P<0.05; **P<0.001.

**Figure 3:**
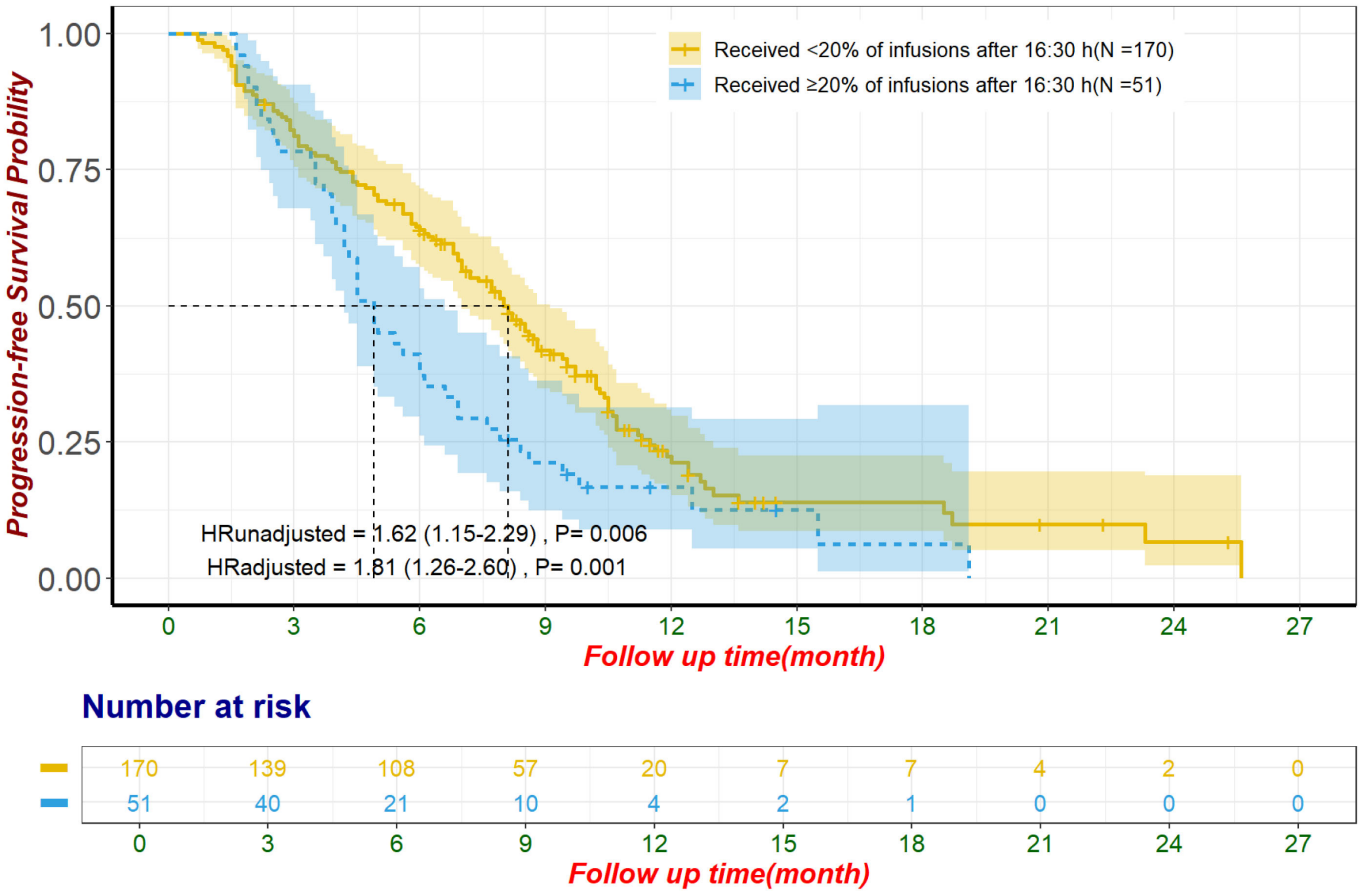
Progression-free survival for unmatched population The adjustment factors include site of origin, degree of differentiation, type of ICI, combination with chemotherapy, line of treatment for ICI, pre-treatment CA199 level less than 500 U/mL, pre-treatment CEA level less than 5 ng/mL, pre-treatment CA125 less than 28.65 U/mL, received subsequent treatment. CA125, Carbohydrate Antigen 125; CA19-9, Carbohydrate Antigen 19-9; CEA, Carcinoembryonic Antigen; ICI, immune checkpoint inhibitor.

In the unmatched cohort, patients with ≥20% of ICI infusions after 16:30 had significantly reduced PFS, with a median of 4.9 months (95% CI: 4.2–6.6) compared to 8.1 months (95% CI: 7.0–9.1) for those with fewer late infusions (HR: 1.62, 95% CI: 1.15–2.29, p = 0.006; **Figure 3**). Multivariate Cox regression confirmed this result, adjusting for influential factors (HR: 1.81, 95% CI: 1.26–2.60, p = 0.001; **Figure 3**). No significant differences were found in ORR (χ²=1.53, p = 0.215; **Table 2**) or in AE occurrences, including Grade 3/4 AE, irAEs, and discontinuations due to AE (all p > 0.050; **Table 3**). The most common irAEs observed included thyroid dysfunction (38 patients, 17.2%), rash (14 patients, 6.3%), and cardiac events (11 patients, 5.0%).

**Table 2:** ORR comparison in the unmatched population who did (n=51) and did not (n=170) receive ≥20% of infusions after 1630 h.

Group	ORR, n(%)		χ^2^	*P* value
	Yes	No		
Percentage infusions after 16:30h≥20%	8 (16)	43 (84)	1.53	0.215
Percentage infusions after 16:30h<20%	43 (25)	127 (75)		

**Table 3:** Adverse events comparison in the unmatched population who did (n=51) and did not (n=170) receive ≥20% of infusions after 1630 h.

AE	Unmatched population		*P* value
	Percentage infusions after 16:30h≥20%(n=51)	Percentage infusions after 16:30h<20%(n=170)	
AE	51(100)	160(94)	0.122^1^
Grade 3/4 AE	23(45)	70(41)	0.619^2^
irAE	20(39)	59(35)	0.556^2^
Grade 3/4 irAE	5(10)	14(8)	0.777^1^
AE leading to discontinuation of medication	5(10)	17(10)	0.967^2^

AE, Adverse event; irAE, Immune-related adverse event.

1. Fisher exact probability method; 2. Chi-square test.

We further analyzed the impact of ICI infusion timing using 16:00 and 15:30 as cutoffs. In the propensity score-matched sample, multivariate Cox regression showed no significant association with OS at the 16:00 cutoff (HR: 1.23, 95% CI: 0.82-1.84, p = 0.321) and the 15:30 cutoff (HR: 1.26, 95% CI: 0.86-1.86, p = 0.237; **Supplementary Table S3**). In the unmatched sample, ICI timing was significantly associated with PFS, with HRs of 1.55 (95% CI: 1.11–2.16, p = 0.009) for 16:00 and 1.71 (95% CI: 1.24-2.37, p = 0.001) for 15:30 (**Supplementary Table S4**) after adjustment in multivariate Cox proportional hazards regression. No significant differences in ORR or irAEs, including Grade 3/4, were observed for either cutoff (**Supplementary Tables S5**, **S6**).

Additional analyses on patients with ≥3 ICI infusions showed that later infusion times were significantly associated with poorer OS after propensity score matching and multivariate Cox regression adjustment, using 16:30, 16:00, and 15:30 as cutoffs (**Supplementary Table S3**). In the unmatched cohort, late infusion times were also significantly linked to reduced PFS at all three cutoffs after multivariate Cox regression adjustment (**Supplementary Table S4**). No significant associations were found between infusion timing and ORR, incidence of irAEs, or Grade 3/4 irAEs at any cutoff (**Supplementary Tables S5**–**S7**).

**Figure 4:**
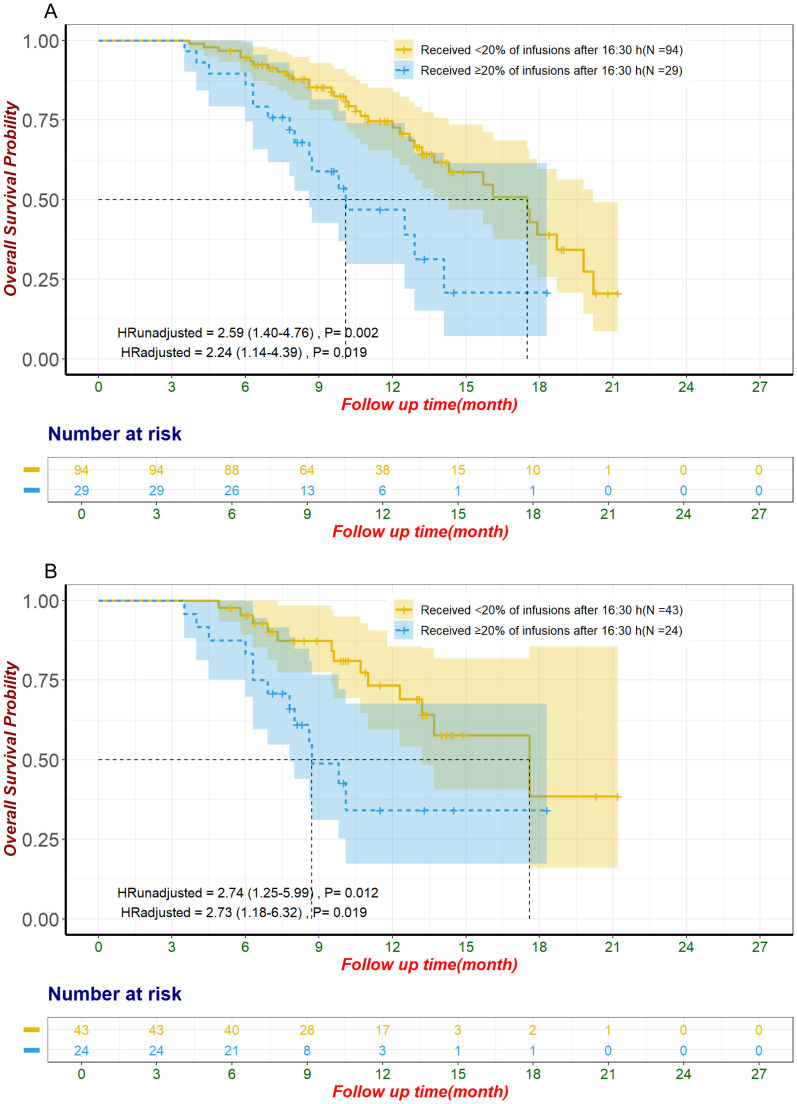
Overall survival in patients receiving first-line ICI plus chemotherapy for unmatched groups **(A)** and propensity score-matched groups **(B)**. The adjustment factors include virology status, disease status, degree of differentiation, pre-treatment CA19-9 level less than 500 U/mL, pre-treatment CEA level less than 5 ng/mL, pre-treatment CA125 level less than 28.65 U/mL, use of antibiotics within one month after immunization. CA125, Carbohydrate Antigen 125; CA19-9, Carbohydrate Antigen 19-9; CEA, Carcinoembryonic Antigen; ICI, immune checkpoint inhibitor.

**Figure 5:**
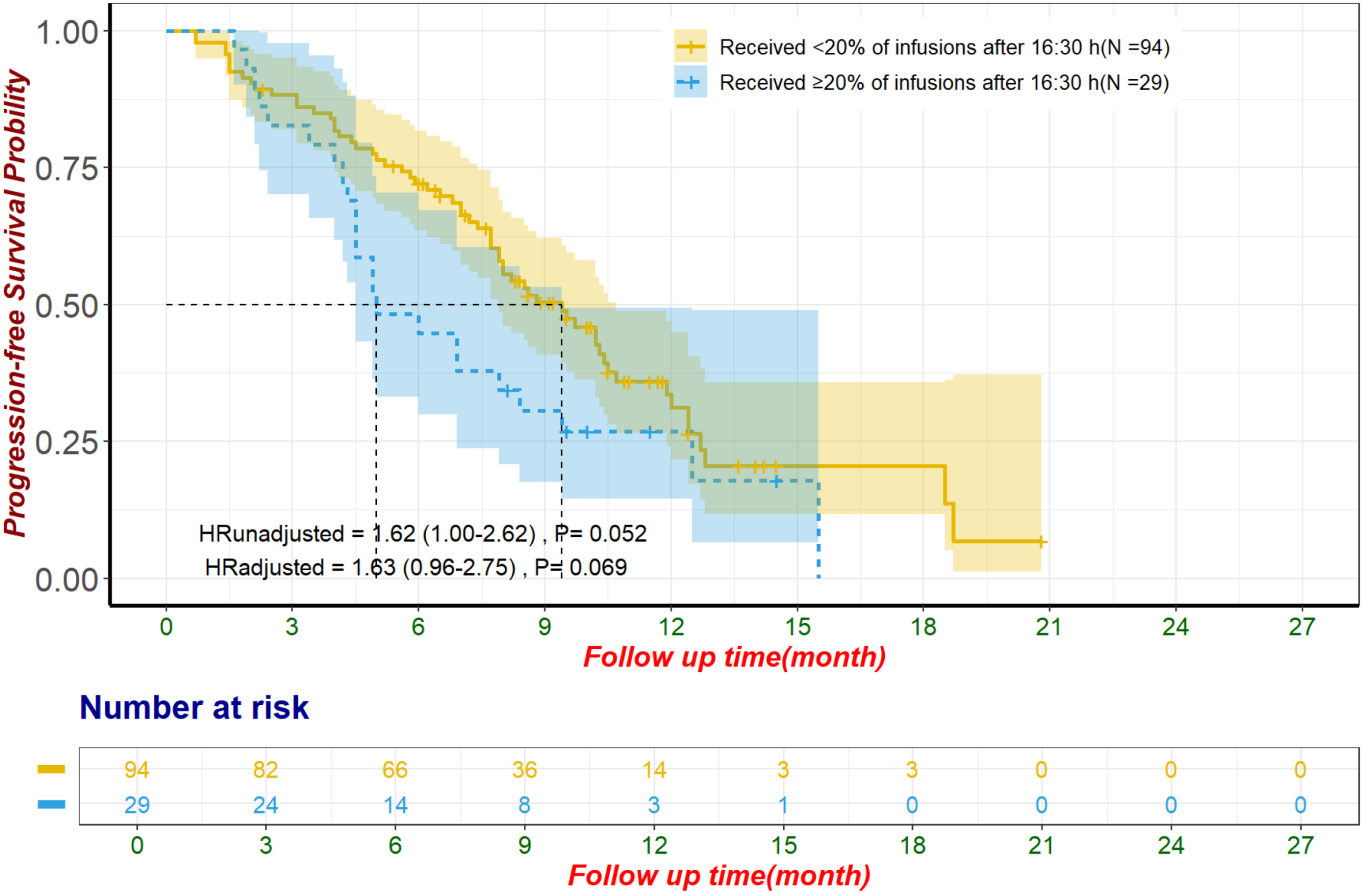
Progression-free survival in patients receiving first-line ICI plus chemotherapy for unmatched population The adjustment factors include site of origin, pre-treatment CA199 level less than 500 U/mL, pre-treatment CA125 less than 28.65 U/mL, received subsequent treatment. CA125, Carbohydrate Antigen 125; CA19-9, Carbohydrate Antigen 19-9; ICI, immune checkpoint inhibitor.

To enhance the clinical relevance of our findings and reduce the impact of inter-patient heterogeneity, we conducted a subgroup analysis focusing on patients receiving first-line ICI plus chemotherapy. This subgroup included 123 patients, of whom 29 (24%) received ≥20% of their ICI infusions after 16:30, while 94 (76%) did not. The baseline characteristics of these patients are summarized in **Supplementary Table S8**. In the unmatched cohort, patients with ≥20% of ICI infusions after 16:30 had significantly shorter OS (median OS: 10.1 months, 95% CI: 8.6–not estimable) compared to those with <20% of ICI infusions after this time (median OS: 17.5 months, 95% CI: 14.3–20.2) (HR: 2.59, 95% CI: 1.40–4.76, p = 0.002, **Figure 4**). This difference remained significant after adjustment using multivariate Cox regression (HR: 2.24, 95% CI: 1.14–4.39, p = 0.019). Following propensity score matching, 67 patients were included in the matched cohort, with 24 patients (36%) receiving ≥20% of ICI infusions after 16:30 and 43 patients (64%) receiving <20%. In the matched cohort, patients with later infusions had significantly shorter OS (median OS: 8.7 months, 95% CI: 7.8–not estimable) compared to those with earlier infusions (median OS: 17.6 months, 95% CI: 13.2–not estimable) (HR: 2.74, 95% CI: 1.25–5.99, p = 0.012, **Figure 4**). This finding remained robust after adjustment using multivariate Cox regression (HR: 2.73, 95% CI: 1.18–6.32, p = 0.019). In contrast, PFS showed a nonsignificant trend toward poorer outcomes in patients with ≥20% of infusions after 16:30 in the unmatched cohort (median PFS: 5.0 months, 95% CI: 4.5–9.4) compared to those with <20% (median PFS: 9.4 months, 95% CI: 7.9–10.7) (HR: 1.62, 95% CI: 1.00–2.62, p = 0.052, **Figure 5**). This trend did not reach statistical significance after multivariate Cox regression adjustment (HR: 1.63, 95% CI: 0.96–2.75, p = 0.069). For the alternative cutoff points of 16:00 and 15:30, no significant differences in OS were observed in the matched cohort, nor were there significant differences in PFS in the unmatched cohort (**Supplementary Table S9**). No significant differences were observed in ORR or irAEs, including Grade 3/4 events, for either cutoff (**Supplementary Tables S10**). Considering the small sample size, no further subgroup analysis was conducted for patients who received ≥3 ICI infusions.

## 4 Discussion

Our study found that administering ≥20% of ICI infusions after 16:30 was linked to poorer OS and PFS in advanced BTC patients, without significantly affecting ORR or AE incidence. This is the first study to explore the impact of chronotherapy on ICI efficacy in BTC, suggesting that infusion timing may crucially influence outcomes. These findings highlight the potential for optimizing ICI administration timing in clinical practice to enhance prognosis for advanced BTC patients.

Our study aligns with the limited body of research on the chronotherapy of ICIs in cancer patients, which has predominantly focused on malignancies such as melanoma (10, 16), non-small cell lung cancer (12, 15), and renal cell carcinoma (13)—cancers relatively more responsive to immunotherapy. In contrast, BTC, which are highly malignant with limited treatment options, have only recently begun to explore the potential of immunotherapy, with limited experience in its use. The standard first-line treatment for advanced BTC has long been the gemcitabine plus cisplatin regimen, as established by the ABC-02 study (3), with no standard second-line treatments prior to the 2021 ABC-06 study (24), leading to generally poor prognoses. BTC is typically considered an immunologically “cold” tumor (19–21), and the effectiveness of ICIs in this context has been disappointing. Apart from a select group of patients with high microsatellite instability (MSI-H)/mismatch repair deficiency (dMMR) or high PD-L1 expression (25), the efficacy of ICIs remains limited. Data from various small single-arm studies indicate that, for the majority of BTC patients, ORR to ICIs ranges from 3% to 13%, and OS is between 5.2 to 8.1 months (26, 27). It was not until the recent publications of the TOPAZ-1 and KEYNOTE-966 studies that ICIs were formally incorporated into the first-line treatment for advanced BTC, though the improvement in survival has been marginal—durvalumab in the TOPAZ-1 study increased median OS from 11.5 months to 12.8 months, and pembrolizumab in the KEYNOTE-966 study from 10.9 months to 12.7 months (4, 5). Given the limited clinical experience with ICIs in advanced BTC, optimizing treatment modalities and identifying effective prognostic biomarkers or strategies to enhance ICI efficacy remain critical. Our findings indicate that the timing of ICI infusion significantly influences patient outcomes, suggesting that adjusting ICI administration times—a cost-effective intervention—could potentially enhance the effectiveness of immunotherapy in clinical practice for patients with advanced BTC.

BTCs are uncommon (1), and the recent introduction of immunotherapy in this area has limited the available research samples. In our retrospective study, we did not restrict variables like the line of immunotherapy to maximize sample size but used statistical techniques to balance confounding factors. Nevertheless, in the subgroup of patients receiving first-line chemotherapy plus ICI, a positive result was still observed, with ≥20% of ICI infusions after 16:30 being significantly associated with poorer OS. The absence of randomization in retrospective studies may lead to uneven factor distribution, potentially skewing results. To improve robustness, we included a wide range of confounders and identified key variables affecting OS during univariate analysis. Given the small sample size and the risk of reduced testing power and collinearity with multiple covariates in multivariate Cox regression, we used propensity score matching to compare patients across two treatment groups. This method is particularly useful in studies with multiple covariates (28). A 1:2 matching ratio increased the overall sample size. We also explored the effects of different ICI infusion cutoffs (15:30 and 16:00) on survival outcomes; however, not both times revealed significant differences in OS, suggesting that critical periods affecting ICI efficacy need further exploration. The decision to use 16:30 as the cutoff was based on more definitive evidence of its impact (10, 12, 13, 15). Previous studies selected this time not based on theoretical considerations but for practical reasons, such as facilitating reasonable grouping and clinical implementation (10). Similarly, while the MEMOIR study also conducted sensitivity analyses with different time cutoffs, no significant differences in OS were found when using 16:00 as a boundary (10). Although other studies have reported variations with different cutoffs (11, 16, 17), they generally suffer from small sample sizes and lack robust statistical validation. For patients receiving three or more ICI infusions, later infusion timing significantly correlated with reduced OS across all evaluated cutoffs. This discrepancy may be due to fewer observations and more confounders in those with fewer infusions. In fact, the MEMOIR study similarly limited its analysis to patients with at least four infusions. Additionally, categorizing patients solely by infusion timing may not precisely assess ICI administration timing, as it doesn’t account for variations among patients within the same group. Unlike OS, later ICI infusion was consistently linked to poorer PFS across cutoffs and infusion counts, likely due to fewer censored cases and less susceptibility to external confounders. In the subgroup of patients receiving first-line ICI plus chemotherapy, later ICI infusion showed a trend toward poorer PFS, but this did not reach statistical significance, potentially due to the relatively small sample size of this subgroup. No link was found between the timing of ICI infusion and the ORR, the incidence of irAEs or Grade 3/4 irAEs. The low incidence of these outcomes calls for further investigation with larger datasets. Regardless, future prospective trials with randomized infusion timings and larger sample sizes are crucial for validating these findings.

Our study differs from similar research like the MEMOIR study, which required a minimum of four ICI infusions for inclusion (10). We set a lower threshold of two infusions, mainly because in China, patients typically undergo imaging and efficacy evaluations after two to three cycles of systemic treatment. If tumor progression is observed at this stage, treatment plans are often adjusted, and ICI may be discontinued. Excluding patients with fewer than four infusions could omit a significant subset who stop ICI due to apparent poor response, potentially skewing the representation of ICI therapy’s impact in advanced BTC. A four-infusion threshold would also reduce our sample size, weakening the study’s statistical power. Including patients with at least two infusions is thus justified. To enhance robustness, we conducted exploratory analyses on patients receiving at least three infusions, showing consistent positive results across all time points—16:30, 16:00, and 15:30. In contrast, Rousseau et al.’s study (12) on non-small cell lung cancer included the number of ICI infusions as a covariate in their Cox regression and found no correlation between infusion timing and prognosis. However, the number of infusions reflects both treatment efficacy and patient survival; better responses and longer survival often lead to continued ICI, while poor responses result in early cessation. Including infusion number as a covariate may obscure the true effects of infusion timing on outcomes, so we excluded it from our models. Additionally, a pan-cancer retrospective study involving diverse cancers found no significant differences in PFS or OS between patients who received over 20% of their pembrolizumab infusions after 16:30 compared to those who did not (14). This study’s mixed nature, with over 30% rare solid tumors, introduces high variability in tumor immunogenicity, complicating the assessment of ICI infusion timing’s true impact on outcomes across cancers.

Human circadian rhythms may influence ICI efficacy based on treatment timing, paralleling established effects seen in chemotherapy and radiotherapy (29, 30). Studies indicate morning chemotherapy improves prognosis and tolerance (31, 32), and cancer cell radiosensitivity varies by time, with better outcomes from morning treatments (30, 33, 34). These variations likely arise from daily oscillations in metabolic proteins, including those involved in oxidation/reduction reactions and xenobiotic clearance, suggesting optimal treatment timing enhances efficacy and reduces adverse effects (35–37). Immunotherapy timing was largely overlooked until the MEMOIR study highlighted its potential impact (10). Both innate and adaptive immune activities exhibit circadian fluctuations (38, 39). For example, the migration of lymphocytes and dendritic cells to lymph nodes peaks at night, and these cells tend to leave during the day (40–42), and vaccinations later in the day elicit weaker adaptive responses (43, 44). Similarly, the levels of CD4+ and CD8+ T cells are lowest around 16:00, potentially reducing adaptive responses (45, 46). In mice, interferon-β shows stronger antitumor effects during the day (8). IL-2 timing affects survival in renal cell carcinoma patients, favoring morning or nighttime doses (9). A recent Nature study found dendritic cells migrate rhythmically to lymph nodes, impacting CD8+ T cell responses and immunotherapy outcomes (42). Additionally, rhythmic PD-1 expression in tumor-associated macrophages was noted, with peak expression enhancing PD-1 inhibitor efficacy (47). Although ICIs have long half-lives (48), potentially diminishing timing impact, observations suggest a nuanced interaction. A plausible explanation could involve the different pharmacokinetics of ICIs in the blood versus the tumor microenvironment (TME). One reason solid tumors respond less effectively to systemic treatments compared to hematological cancers is the limited direct exposure of drugs within the TME (49). Despite the prolonged half-life of ICIs in the bloodstream, their behavior in the TME can be markedly different. For instance, Cancer cells may secrete immune checkpoint fragments that neutralize ICIs, reducing efficacy (50, 51). If ICIs are given when TME immune cells are inactive, these drugs might be progressively captured by secreted immune checkpoint fragments. By the time immune cells reactivate, the concentration of ICIs may no longer be sufficient to effectively stimulate anti-tumor immunity. Nonetheless, these underlying mechanisms still require further experimental exploration to be definitively understood. For a comprehensive understanding of circadian rhythms and their impact on immunotherapy, recent reviews that detail the underlying biological mechanisms can be highly informative (52–54).

Our study has limitations. Firstly, as a single-center retrospective study, there is an inherent risk of bias, even with propensity score matching to balance confounders. Secondly, the rarity of BTC and the recent use of ICIs limited our sample size. We did not restrict immunotherapy lines or combination regimens, which may have introduced bias, though propensity score matching was used to minimize this. Additionally, we did not analyze factors like genetic mutations, PD-L1 expression, tumor mutational burden, or microsatellite instability due to limited testing. Future large, multicenter randomized trials are needed to validate these findings, alongside basic research to clarify how ICI timing affects efficacy.

In summary, our findings indicate that the timing of ICI infusions significantly affects the efficacy of immunotherapy in patients with advanced BTC. Patients receiving their ICI infusions before 16:30 demonstrated improved survival outcomes compared to those who received treatments later. Given these findings, it may be prudent to consider earlier ICI infusions in the daily treatment schedule for patients with advanced BTC, to potentially enhance therapeutic outcomes.

## Data Availability

The raw data supporting the conclusions of this article will be made available by the authors, without undue reservation.
