# Supplementary material for: Effect of immune checkpoint inhibitor time-of-day infusion on survival in advanced biliary tract cancer: a propensity score-matched analysis
Source: Front Immunol. 2024 Dec 18;15:1512972. doi: 10.3389/fimmu.2024.1512972 (PMC11688298; doi:10.3389/fimmu.2024.1512972)
Supplement: Supplementary file 1 [file Table1.docx]

| **Table S1. Patient characteristics in the** **unmatched and propensity score-matched population** | | | | | | |
| --- | --- | --- | --- | --- | --- | --- |
| **Variate** | **Unmatched population** | | ***P*** value | **Matched population** | | ***P*** value |
|  | **Received ≥20% of infusions after 1630 h**(n=51) | **Received <20% of infusions after 1630 h**(n=170) |  | **Received ≥20% of infusions after 1630 h**(n=49) | **Received <20% of infusions after 1630 h**(n=90) |  |
| Sex,n(%) | | | | | | |
| Male | 21(41) | 96(56) | 0.05^1*^ | 21(43) | 55(61) | 0.04^1*^ |
| Female | 30(59) | 74(44) |  | 28(57) | 35(39) |  |
| Age(years),Mean±SD | 56.31±11.31 | 58.47±9.91 | 0.22^2^ | 56.29±11.48 | 57.87±9.87 | 0.58^2^ |
| Virology status,n(%) | | | | | | |
| No viral hepatitis | 18(35) | 50(29) | 0.61^3^ | 18(37) | 23(26) | 0.18^3^ |
| Any viral hepatitis B | 33(65) | 119(70) |  | 31(63) | 67(74) |  |
| Prior hepatitis C | 0(0) | 1(1) |  | 0(0) | 0(0) |  |
| Disease status,n(%) | | | | | | |
| Initially unresectable | 31(61) | 79(46) | 0.07^1^ | 31(63) | 40(44) | 0.03^1*^ |
| Recurrent | 20(39) | 91(54) |  | 18(37) | 50(56) |  |
| Disease classification,n(%) | | | | | | |
| Locally advanced | 8(16) | 36(21) | 0.39^1^ | 8(16) | 20(22) | 0.41^1^ |
| Metastatic | 43(84) | 134(79) |  | 41(84) | 70(78) |  |
| Site of origin,n(%) | | | | | | |
| Intrahepatic | 33(65) | 109(64) | 0.67^1^ | 33(67) | 65(72) | 0.68^1^ |
| Perihilar | 5(10) | 21(12) |  | 5(10) | 11(12) |  |
| Distal | 2(4) | 13(8) |  | 2(4) | 4(4) |  |
| Gallbladder | 11(22) | 27(16) |  | 9(18) | 10(11) |  |
| Degree of differentiation,n(%) | | | | | | |
| Poorly | 32(63) | 105(62) | 0.90^1^ | 31(63) | 59(66) | 0.79^1^ |
| moderately-to-well | 19(37) | 65(38) |  | 18(37) | 31(34) |  |
| Type of ICI,n(%) | | | | | | |
| Anti-PD-1 | 35(69) | 113(66) | 0.77^1^ | 33(67) | 61(68) | 0.96^1^ |
| Anti-PD-L1 | 16(31) | 57(34) |  | 16(33) | 29(32) |  |
| ICI,n(%) | | | | | | |
| Durvalumab | 12(24) | 47(28) | 0.12^3^ | 12(24) | 24(27) | 0.41^3^ |
| Pembrolizuma | 1(2) | 19(11) |  | 1(2) | 6(7) |  |
| Sintilimab | 15(29) | 35(21) |  | 13(27) | 19(21) |  |
| Camrelizumab | 5(10) | 20(12) |  | 5(10) | 11(12) |  |
| Toripalimab | 8(16) | 12(7) |  | 8(16) | 6(7) |  |
| others | 10(20) | 37(22) |  | 10(20) | 24(27) |  |
| Combination with chemotherapy,n(%) | | | | | | |
| no | 7(14) | 22(13) | 0.88^1^ | 7(14) | 12(13) | 0.88^1^ |
| Yes | 44(86) | 148(87) |  | 42 (86) | 78(87) |  |
| Combination with anti-angiogenic drugs,n(%) | | | | | | |
| no | 40(78) | 140(82) | 0.53^1^ | 38(78) | 76(84) | 0.31^1^ |
| yes | 11(22) | 30(18) |  | 11(22) | 14(16) |  |
| Combined with other targeted drugs,n(%) | | | | | | |
| no | 50(98) | 167(98) | 1.00^3^ | 48(98) | 89(99) | 1.00^3^ |
| yes | 1(2) | 3(2) |  | 1(2) | 1(1) |  |
| Line of treatment for ICI,n(%) | | | | | | |
| First line | 33(65) | 104(61) | 0.65^1^ | 32(65) | 60(67) | 0.87^1^ |
| ≥2 lines | 18(35) | 66(39) |  | 17(35) | 30(33) |  |
| ECOG performance status,n(%) | | | | | | |
| 0 | 32(63) | 133(78) | 0.03^1*^ | 30(61) | 75(83) | 0.004^1*^ |
| ≥1 | 19(37) | 37(22) |  | 19(39) | 15(17) |  |
| Have received radiotherapy,n(%) | | | | | | |
| no | 42(82) | 126(74) | 0.23^1^ | 40(82) | 66(73) | 0.27^1^ |
| yes | 9(18) | 44(26) |  | 9(18) | 24(27) |  |
| Have undergone interventional therapy,n(%) | | | | | | |
| no | 36(71) | 129(76) | 0.45^1^ | 34(69) | 67(74) | 0.52^1^ |
| yes | 15(29) | 41(24) |  | 15(31) | 23(26) |  |
| Pre-treatment CA19-9<500 U/mL,n(%) | | | | | | |
| no | 14(27) | 51(30) | 0.73^1^ | 14(29) | 23(26) | 0.70^1^ |
| yes | 37(73) | 119(70) |  | 35(71) | 67(74) |  |
| Pre-treatment CEA<5 ng/mL,n(%) | | | | | | |
| no | 20(39) | 62(36) | 0.72^1^ | 18(37) | 33(37) | 1.00^1^ |
| yes | 31(61) | 108(64) |  | 31(63) | 57(63) |  |
| Pre-treatment CA125<28.65 U/mL,n(%) | | | | | | |
| no | 26(51) | 89(52) | 0.86^1^ | 26(53) | 40(44) | 0.33^1^ |
| yes | 25(49) | 81(48) |  | 23(47) | 50 (56) |  |
| NLR≤3,n(%) | | | | | | |
| no | 28(55) | 82(48) | 0.40^1^ | 28(57) | 50(56) | 0.86^1^ |
| yes | 23(45) | 88(52) |  | 21(43) | 40(44) |  |
| Received subsequent treatment,n(%) | | | | | | |
| no | 24(47) | 100(59) | 0.14^1^ | 24(49) | 51(57) | 0.39^1^ |
| yes | 27(53) | 70(41) |  | 25(51) | 39(43) |  |
| Use of antibiotics within one month after immunization,n(%) | | | | | | |
| no | 49(96) | 162(95) | 1.00^3^ | 47(96) | 84(93) | 0.71^3^ |
| yes | 2(4) | 8(5) |  | 2(4) | 6(7) |  |
| Smoke,n(%) | | | | | | |
| Never | 39(76) | 131(77) | 0.93^1^ | 38(78) | 67(74) | 0.68^1^ |
| Former/Current | 12(24) | 39(23) |  | 11(22) | 23(26) |  |
| ICI, immune checkpoint inhibitor; ECOG, Eastern Cooperative Oncology Group; CA19-9, Carbohydrate Antigen 19-9; CEA, Carcinoembryonic Antigen; CA125, Carbohydrate Antigen 125; NLR, Neutrophil-to-Lymphocyte Ratio  1.Chi-square test;2.t test;3.Fisher's Exact Test.**P≤*0.05 | | | | | | |
