# Supplementary material for: Effect of immune checkpoint inhibitor time-of-day infusion on survival in advanced biliary tract cancer: a propensity score-matched analysis
Source: Front Immunol. 2024 Dec 18;15:1512972. doi: 10.3389/fimmu.2024.1512972 (PMC11688298; doi:10.3389/fimmu.2024.1512972)
Supplement: Supplementary file 2 [file Table2.docx]

| **Table S2.** Univariate Cox proportional hazard regression for overall survival in an unmatched population with two infusions(n=221) | | | |
| --- | --- | --- | --- |
| **Covariate** | **Overall Survival HR** | **HR 95%CI** | ***P* value** |
| Female | 0.932 | 0.655~1.33 | 0.698 |
| Age | 1.01 | 0.992~1.03 | 0.326 |
| Virology status(Reference=No viral hepatitis) | | | |
| Any viral hepatitis B | 0.941 | 0.645~1.37 | 0.754 |
| Prior hepatitis C | 3.54 | 0.481~26.0 | 0.215 |
| Disease status(Reference=Initially unresectable) | | | |
| Recurrent | 1.02 | 0.713~1.46 | 0.920 |
| Disease classification(Reference=Locally advanced) | | | |
| Metastatic | 1.47 | 0.916~2.36 | 0.110 |
| Site of origin(Reference=Intrahepatic) | | | |
| Perihilar | 1.73 | 1.01-2.96 | 0.045* |
| Distal | 1.47 | 0.73-2.96 | 0.277 |
| Gallbladder | 1.72 | 1.13~2.63 | 0.012* |
| Degree of differentiation(Reference=Poorly) | | | |
| moderately-to-well | 0.648 | 0.448~0.939 | 0.022* |
| Type of ICI(Reference=Anti-PD-1) | | | |
| Anti-PD-L1 | 0.516 | 0.34~0.782 | 0.002* |
| Combination with chemotherapy | 0.854 | 0.531~1.38 | 0.517 |
| Combination with anti-angiogenic drugs | 1.15 | 0.749~1.76 | 0.528 |
| Combined with other targeted drugs | 1.48 | 0.469~4.66 | 0.505 |
| Line of treatment for ICI(≥2 lines) | 2.12 | 1.48~3.03 | 3.67e-5* |
| ECOG performance status≥1 | 1.23 | 0.839~1.81 | 0.287 |
| Have received radiotherapy | 0.859 | 0.571~1.29 | 0.467 |
| Have undergone interventional therapy | 0.727 | 0.48~1.1 | 0.134 |
| Pre-treatment CA19-9<500 U/mL | 0.419 | 0.29~0.607 | 4.16e-6* |
| Pre-treatment CEA<5 ng/mL | 0.523 | 0.366~0.747 | 0.00036* |
| Pre-treatment CA125<28.65 U/mL | 0.467 | 0.321~0.679 | 6.56e-5* |
| NLR≤3 | 0.759 | 0.533~1.08 | 0.125 |
| Received subsequent treatment | 0.895 | 0.627~1.28 | 0.539 |
| Use of antibiotics within one month after immunization | 1.69 | 0.742~3.86 | 0.211 |
| Smoke | 0.659 | 0.415~1.05 | 0.0765* |
| ICI, immune checkpoint inhibitor; ECOG, Eastern Cooperative Oncology Group; CA19-9, Carbohydrate Antigen 19-9; CEA, Carcinoembryonic Antigen; CA125, Carbohydrate Antigen 125; NLR, Neutrophil-to-Lymphocyte Ratio  **P*<0.10 | | | |
