# Supplementary material for: Effect of immune checkpoint inhibitor time-of-day infusion on survival in advanced biliary tract cancer: a propensity score-matched analysis
Source: Front Immunol. 2024 Dec 18;15:1512972. doi: 10.3389/fimmu.2024.1512972 (PMC11688298; doi:10.3389/fimmu.2024.1512972)
Supplement: Supplementary file 3 [file Table3.docx]

**Table S3.** Sensitivity analysis of propensity score-matched groups with multivariable Cox proportional hazards regression, applied to varying infusion time cutoffs for overall survival

| **Two** **infusions** | **HRadjusted(95%CI)** | ***P* value** |
| --- | --- | --- |
| ≥20% infusions after 15:30 versus <20% infusions after 15:30h | 1.26 (0.86-1.86) | 0.237 |
| ≥20% infusions after 16:00 versus <20% infusions after 16:00h | 1.23 (0.82-1.84) | 0.321 |
| **Three infusions** | **HRadjusted(95%CI)** | ***P* value** |
| ≥20% infusions after 15:30 versus <20% infusions after 15:30h | 2.09 (1.26-3.46) | 0.004* |
| ≥20% infusions after 16:00 versus <20% infusions after 16:00h | 2.18 (1.32-3.62) | 0.003* |
| ≥20% infusions after 16:30 versus <20% infusions after 16:30h | 2.39 (1.26-4.53) | 0.008* |

**P*<0.05；***P≤*0.001
