# Supplementary material for: Effect of immune checkpoint inhibitor time-of-day infusion on survival in advanced biliary tract cancer: a propensity score-matched analysis
Source: Front Immunol. 2024 Dec 18;15:1512972. doi: 10.3389/fimmu.2024.1512972 (PMC11688298; doi:10.3389/fimmu.2024.1512972)
Supplement: Supplementary file 4 [file Table4.docx]

**Table S4.** Sensitivity analysis of unmatched groups with multivariable Cox proportional hazards regression, applied to varying infusion time cutoffs for progression free survival

| **Two infusions** | **HRadjusted(95%CI)** | ***P* value** |
| --- | --- | --- |
| ≥20% infusions after 15:30 versus <20% infusions after 15:30h | 1.71 (1.24-2.37) | 0.001** |
| ≥20% infusions after 16:00 versus <20% infusions after 16:00h | 1.55 (1.11~2.16) | 0.009* |
| **Three infusions** |  |  |
| ≥20% infusions after 15:30 versus <20% infusions after 15:30h | 2.48 (1.70-3.62) | <0.001** |
| ≥20% infusions after 16:00 versus <20% infusions after 16:00h | 2.16 (1.48-3.16) | <0.001** |
| ≥20% infusions after 16:30 versus <20% infusions after 16:30h | 2.77 (1.81-4.25) | <0.001** |

**P*<0.05；***P≤*0.001
