# Supplementary material for: Effect of immune checkpoint inhibitor time-of-day infusion on survival in advanced biliary tract cancer: a propensity score-matched analysis
Source: Front Immunol. 2024 Dec 18;15:1512972. doi: 10.3389/fimmu.2024.1512972 (PMC11688298; doi:10.3389/fimmu.2024.1512972)
Supplement: Supplementary file 5 [file Table5.docx]

**Table S5.** Sensitivity analysis of unmatched groups for objective response rate, applied to varying infusion time cutoffs

| **Two infusions** | **≥20% infusions** | **<20% infusions** | ****$\chi^{2}$ | ***P* value** |
| --- | --- | --- | --- | --- |
| after 15:30h | 17(19.5%) | 34(25.4%) | 1.011 | 0.315 |
| after 16:00h | 12(17.6%) | 39(25.5%) | 1.631 | 0.202 |
| **Three infusions** | **≥20% infusions** | **<20% infusions** | ****$\chi^{2}$ | ***P* value** |
| after 15:30h | 17(22.7%) | 33(32.0%) | 1.887 | 0.170 |
| after 16:00h | 12(20.7%) | 38(31.7%) | 2.333 | 0.127 |
| after 16:30h | 8(19.0%) | 42(30.9%) | 2.225 | 0.136 |

**P*<0.05;
