# Supplementary material for: Effect of immune checkpoint inhibitor time-of-day infusion on survival in advanced biliary tract cancer: a propensity score-matched analysis
Source: Front Immunol. 2024 Dec 18;15:1512972. doi: 10.3389/fimmu.2024.1512972 (PMC11688298; doi:10.3389/fimmu.2024.1512972)
Supplement: Supplementary file 6 [file Table6.docx]

**Table S6.** Immune-related adverse events at different infusion times

| **Two infusions** | **≥20% infusions** | **<20% infusions** | ****$\chi^{2}$ | ***P* value** |
| --- | --- | --- | --- | --- |
| after 15:30h | 34(39.1%) | 45(33.6%) | 0.694 | 0.405 |
| after 16:00h | 28(41.2%) | 51(7.2%) | 1.254 | 0.263 |
| **Three infusions** | **≥20% infusions** | **<20% infusions** | ****$\chi^{2}$ | ***P* value** |
| after 15:30h | 30(40.0%) | 36(35.0%) | 0.474 | 0.491 |
| after 16:00h | 24(41.4%) | 42(35.0%) | 0.682 | 0.409 |
| after 16:30h | 17(40.5%) | 49(36.0%) | 0.272 | 0.602 |
