# Supplementary material for: Effect of immune checkpoint inhibitor time-of-day infusion on survival in advanced biliary tract cancer: a propensity score-matched analysis
Source: Front Immunol. 2024 Dec 18;15:1512972. doi: 10.3389/fimmu.2024.1512972 (PMC11688298; doi:10.3389/fimmu.2024.1512972)
Supplement: Supplementary file 7 [file Table7.docx]

**Table S7.** Grade 3/4 immune-related adverse events at different infusion times

| **Two infusions** | **≥20% infusions** | **<20% infusions** | ****$\chi^{2}$ | ***P* value** |
| --- | --- | --- | --- | --- |
| after 15:30h | 11(12.6%) | 8(6.0%) | 2.990 | 0.084 |
| after 16:00h | 8(11.8%) | 11(7.2%) | 1.254 | 0.263 |
| **Three infusions** | **≥20% infusions** | **<20% infusions** | ****$\chi^{2}$ | ***P* value** |
| after 15:30h | 9(12.0%) | 5(4.9%) | 3.058 | 0.080 |
| after 16:00h | 6(10.3%) | 8(6.7%) | / | 0.389 |
| after 16:30h | 3(7.1%) | 11(8.1%) | / | 1.000 |
