# Supplementary material for: Effect of immune checkpoint inhibitor time-of-day infusion on survival in advanced biliary tract cancer: a propensity score-matched analysis
Source: Front Immunol. 2024 Dec 18;15:1512972. doi: 10.3389/fimmu.2024.1512972 (PMC11688298; doi:10.3389/fimmu.2024.1512972)
Supplement: Supplementary file 8 [file Table8.docx]

| **Table S8.** **Patient characteristics of those receiving first-line ICI plus chemotherapy in the unmatched and propensity score-matched populations** | | | | | | |
| --- | --- | --- | --- | --- | --- | --- |
| **Variate** | **Unmatched population** | | ***P*** value | **Matched population** | | ***P*** value |
|  | **Received ≥20% of infusions after 1630 h**(n=29) | **Received <20% of infusions after 1630 h**(n=94) |  | **Received ≥20% of infusions after 1630 h**(n=24) | **Received <20% of infusions after 1630 h**(n=43) |  |
| Sex,n(%) | | | | | | |
| Male | 13(45) | 52(55) | 0.32^1^ | 9 (38) | 26 (60) | 0.07^1^ |
| Female | 16(55) | 42(45) |  | 15 (63) | 17 (40) |  |
| Age(years),Mean±SD | 58.38±11.92 | 58.31±10.23 | 0.69^2^ | 58.29±10.37 | 57.21±9.17 | 0.57^2^ |
| Virology status,n(%) | | | | | | |
| No viral hepatitis | 12(41) | 23(24) | 0.19^3^ | 8(33) | 13(30) | 0.79^3^ |
| Any viral hepatitis B | 17(59) | 70(74) |  | 16(67) | 30(70) |  |
| Prior hepatitis C | 0(0) | 1(1) |  | 0(0) | 0(0) |  |
| Disease status,n(%) | | | | | | |
| Initially unresectable | 22(76) | 56(60) | 0.11^1^ | 17(71) | 31(72) | 0.91^1^ |
| Recurrent | 7(24) | 38(40) |  | 7(29) | 12(28) |  |
| Disease classification,n(%) | | | | | | |
| Locally advanced | 6(21) | 21(22) | 0.85^1^ | 6 (25) | 8 (19) | 0.54^1^ |
| Metastatic | 23(79) | 73(78) |  | 18 (75) | 35 (81) |  |
| Site of origin,n(%) | | | | | | |
| Intrahepatic | 19(66) | 65(69) | 0.39^1^ | 15 (63) | 34 (79) | 0.075^1^ |
| Perihilar | 2(7) | 14(15) |  | 1 (4) | 5 (12) |  |
| Distal | 2(7) | 5(5) |  | 2 (8) | 1 (2) |  |
| Gallbladder | 6(21) | 10(11) |  | 6 (25) | 3 (7) |  |
| Degree of differentiation,n(%) | | | | | | |
| Poorly | 19(66) | 63(67) | 0.88^1^ | 17(71) | 29(67) | 0.77^1^ |
| moderately-to-well | 10(34) | 31(33) |  | 7(29) | 14(33) |  |
| Type of ICI,n(%) | | | | | | |
| Anti-PD-1 | 15(52) | 48(51) | 0.95^1^ | 13(54) | 21(49) | 0.68^1^ |
| Anti-PD-L1 | 14(48) | 46(49) |  | 11(46) | 22(51) |  |
| ICI,n(%) | | | | | | |
| Durvalumab | 10(34) | 40(43) | 0.35^3^ | 7(29) | 19(44) | 0.68^3^ |
| Pembrolizuma | 1(3) | 9(10) |  | 1(4) | 4(9) |  |
| Sintilimab | 3(10) | 13(14) |  | 3(13) | 5(12) |  |
| Camrelizumab | 3(10) | 7(7) |  | 3(13) | 3(7) |  |
| Toripalimab | 3(10) | 2(2) |  | 1(4) | 2(5) |  |
| others | 9(31) | 23(24) |  | 9(38) | 10(23) |  |
| ECOG performance status,n(%) | | | | | | |
| 0 | 17(59) | 76(81) | 0.015^1*^ | 14(58) | 34(79) | 0.071 |
| ≥1 | 12(41) | 18(19) |  | 10(42) | 9(21) |  |
| Have received radiotherapy,n(%) | | | | | | |
| no | 26(90) | 75(80) | 0.23^1^ | 22(92) | 37(86) | 0.70^1^ |
| yes | 3(10) | 19(20) |  | 2(8) | 6(14) |  |
| Have undergone interventional therapy,n(%) | | | | | | |
| no | 20(69) | 74(79) | 0.28^1^ | 17(71) | 31(72) | 0.91^1^ |
| yes | 9(31) | 20(21) |  | 7(29) | 12(28) |  |
| Pre-treatment CA19-9<500 U/mL,n(%) | | | | | | |
| no | 8(28) | 23(24) | 0.74^1^ | 5(21) | 7(16) | 0.74^1^ |
| yes | 21 (72) | 71 (76) |  | 19(79) | 36(84) |  |
| Pre-treatment CEA<5 ng/mL,n(%) | | | | | | |
| no | 11(38) | 31(33) | 0.62^1^ | 9(37) | 12(28) | 0.42^1^ |
| yes | 18(62) | 63(67) |  | 15(63) | 31(72) |  |
| Pre-treatment CA125<28.65 U/mL,n(%) | | | | | | |
| no | 13(45) | 48(51) | 0.56^1^ | 12(50) | 26(60) | 0.41^1^ |
| yes | 16(55) | 46(49) |  | 12(50) | 17(40) |  |
| NLR≤3,n(%) | | | | | | |
| no | 15(52) | 44(47) | 0.64^1^ | 13(54) | 21(49) | 0.68^1^ |
| yes | 14(48) | 50(53) |  | 11(46) | 22(51) |  |
| Received subsequent treatment,n(%) | | | | | | |
| no | 14(48) | 57(61) | 0.24^1^ | 12 (50) | 23(53) | 0.78^1^ |
| yes | 15(52) | 37(39) |  | 12(50) | 20(47) |  |
| Use of antibiotics within one month after immunization,n(%) | | | | | | |
| no | 27(93) | 88(94) | 0.99^3^ | 22(92) | 42(98) | 0.29^3^ |
| yes | 2(7) | 6(6) |  | 2(8) | 1(2) |  |
| Smoke,n(%) | | | | | | |
| Never | 22(76) | 68(72) | 0.71^1^ | 19(79) | 29(67) | 0.31^1^ |
| Former/Current | 7(24) | 26(28) |  | 5(21) | 14(33) |  |
| ICI, immune checkpoint inhibitor; ECOG, Eastern Cooperative Oncology Group; CA19-9, Carbohydrate Antigen 19-9; CEA, Carcinoembryonic Antigen; CA125, Carbohydrate Antigen 125; NLR, Neutrophil-to-Lymphocyte Ratio  1.Chi-square test;2.t test;3.Fisher's Exact Test.**P≤*0.05 | | | | | | |
