# Supplementary material for: Effect of immune checkpoint inhibitor time-of-day infusion on survival in advanced biliary tract cancer: a propensity score-matched analysis
Source: Front Immunol. 2024 Dec 18;15:1512972. doi: 10.3389/fimmu.2024.1512972 (PMC11688298; doi:10.3389/fimmu.2024.1512972)
Supplement: Supplementary file 9 [file Table9.docx]

**Table S9.** Sensitivity analysis in patients receiving first-line ICI plus chemotherapy: multivariable Cox proportional hazards regression for OS in propensity score-matched groups and for PFS in unmatched groups, applied to varying infusion time cutoffs

| **OS** | **HRadjusted(95%CI)** | ***P* value** |
| --- | --- | --- |
| ≥20% infusions after 15:30 versus <20% infusions after 15:30h | 1.89 (0.99-3.61) | 0.055 |
| ≥20% infusions after 16:00 versus <20% infusions after 16:00h | 1.83 (0.92-3.65) | 0.086 |
| **PFS** | **HRadjusted(95%CI)** | ***P* value** |
| ≥20% infusions after 15:30 versus <20% infusions after 15:30h | 1.35 (0.86-2.12) | 0.186 |
| ≥20% infusions after 16:30 versus <20% infusions after 16:00h | NA* | NA* |

ICI, immune checkpoint inhibitor; OS, overall survival; PFS, progression-free survival

*As the univariate Cox proportional hazards regression analysis yielded a p-value of 0.328, this infusion time cutoff was not included in the multivariate analysis.
