# Supplementary material for: Effect of immune checkpoint inhibitor time-of-day infusion on survival in advanced biliary tract cancer: a propensity score-matched analysis
Source: Front Immunol. 2024 Dec 18;15:1512972. doi: 10.3389/fimmu.2024.1512972 (PMC11688298; doi:10.3389/fimmu.2024.1512972)
Supplement: Supplementary file 10 [file Table10.docx]

**Table S10.** Immune-related adverse events, Grade 3/4 immune-related adverse events, and objective response rates at different infusion times in patients receiving first-line ICI plus chemotherapy

| **Immune-related adverse events** | **≥20% infusions** | **<20% infusions** | $\chi^{2}$ | ***P* value** |
| --- | --- | --- | --- | --- |
| after 15:30h | 19(36.5%) | 17(23.9%) | 1.732 | 0.188 |
| after 16:00h | 15(40.5%) | 21(24.4%) | 2.516 | 0.113 |
| after 16:30h | 12(41.4%) | 24(25.5%) | 1.978 | 0.160 |
| **Grade 3/4 immune-related adverse events** | **≥20% infusions** | **<20% infusions** | $\chi^{2}$ | ***P* value** |
| after 15:30h | 7(13.5%) | 4(5.6%) | 1.400 | 0.237 |
| after 16:00h | 4(10.8%) | 7(8.1%) | 0.017 | 0.895 |
| after 16:30h | 2(6.9%) | 9(9.6%) | 0.005 | 0.945 |
| **Objective response rate** | **≥20% infusions** | **<20% infusions** | $\boldsymbol{\chi}^{\mathbf{2}}$ | **P value** |
| after 15:30h | 13(25.0%) | 22(31.0%) | 0.275 | 0.600 |
| after 16:00h | 9(24.3%) | 26(30.2%) | 0.201 | 0.654 |
| after 16:30h | 5(17.2%) | 30(31.9%) | 1.679 | 0.195 |

ICI, immune checkpoint inhibitor
